# A Review of Bioplastics and Their Adoption in the Circular Economy

**DOI:** 10.3390/polym13081229

**Published:** 2021-04-10

**Authors:** Alberto Di Bartolo, Giulia Infurna, Nadka Tzankova Dintcheva

**Affiliations:** Dipartimento di Ingegneria, Università degli Studi di Palermo, Viale delle Scienze, Ed. 6, 90128 Palermo, Italy; alberto.dibartolo@gmail.com (A.D.B.); giulia.infurna@unipa.it (G.I.)

**Keywords:** bioplastic, bio-based plastic, biodegradable plastic, bioeconomy, life cycle assessment, sustainability

## Abstract

The European Union is working towards the 2050 net-zero emissions goal and tackling the ever-growing environmental and sustainability crisis by implementing the *European Green Deal*. The shift towards a more sustainable society is intertwined with the production, use, and disposal of plastic in the European economy. Emissions generated by plastic production, plastic waste, littering and leakage in nature, insufficient recycling, are some of the issues addressed by the European Commission. Adoption of bioplastics–plastics that are biodegradable, bio-based, or both–is under assessment as one way to decouple society from the use of fossil resources, and to mitigate specific environmental risks related to plastic waste. In this work, we aim at reviewing the field of bioplastics, including standards and life cycle assessment studies, and discuss some of the challenges that can be currently identified with the adoption of these materials.

## 1. Introduction

The European Green Deal [1] is the action plan outlined by the European Commission (EC) to tackle the ever-growing environment and climate-related challenges our society faces. The plan aims at transforming *“the EU into a fair and prosperous society, with a modern, resource-efficient and competitive economy where there are no net emissions of greenhouse gases in 2050 and where economic growth is decoupled from resource use”* [1] (p. 2). As also stated in its communication *“A new Circular Economy Action Plan for a Cleaner and More Competitive Europe”* [2], the EC underlines the utmost importance of shifting towards a circular economy, with a framework of policies that make sustainable products, services, and business models the norm. On the global scale, The United Nations Development Program (UNDP) also stressed the importance of working towards a sustainable economy, with efficient use of natural resources, little to none waste and pollution [3]. In this context, the EC identifies a series of pressing challenges relating to plastic production, (mis-)use and pollution, spanning from single-use items, over-packaging, and littering, to microplastics, high-carbon footprints, and lack of appropriate labeling. The strategy outlined to tackle these challenges includes supporting the bio-based industry and developing a framework for the use of bio-based plastics, *“based on assessing where the use of bio-based feedstock results in genuine environmental benefits”*, and for the use of biodegradable or compostable plastics, *“based on an assessment of the applications where such use can be beneficial to the environment”* [2] (p. 9). These plastics, which are either bio-based or biodegradable (or both), are referred to as “bioplastics” and have been the topic of much work and discussion at a global level for some time now. The dwindling of fossil resources provides a strong drive to the development of bio-based products, while the possibility to mitigate environmental pollution or simplify organic waste collection are big motivations behind the development of biodegradable and compostable plastic products. Bioplastics already find applications on the market, particularly as packaging [4,5,6,7], carrier and compost bags [5,6]; they are also applied in the agriculture and horticulture sector [6,8], and in the automotive and electronic industry [6,9]. Furthermore, biodegradable polymers have been long applied in biomedicine [5,10,11]. Still, the production of bio-based plastics is limited to one percent of the worldwide plastic production [7,12] and their adoption comes with uncertainties, as acknowledged in the EC Communication *“A European Strategy for Plastics in a Circular Economy”* [13]. This is exemplified by the research focused on bioplastics sustainability and [8,14,15,16,17,18,19,20,21,22,23,24,25] biodegradability [26,27,28,29,30], as well as the attention of media to the subject. Excluding the ample literature on biomedical applications, academic research has been focusing on the synthesis of bio-based polymers [31,32], on the life cycle assessment (LCA) of the production and end-of-life (EOL) [20,24,33,34] of different bioplastics, and biodegradation under different conditions [10,14,35].

In this paper, we present the reader with an overview of the bioplastics field, including definitions, polymers on the market, and applications. We discuss biotic and abiotic degradation mechanisms and present standards and certifications that are in place to evaluate the compostability and biodegradability of bioplastics. Recent works on the biodegradability of bioplastics are also reviewed. We report on the standards in place for the LCA of bioplastics and review recent studies on the subject, with particular focus on studies that consider the EOL assessment. Finally, given the material reviewed, we concisely discuss the challenges that can be identified with the adoption of bioplastics, as well as possible solutions, and we draw our conclusions on the topic.

### 1.1. Environmental Impact of Plastics

The generation of plastic waste and subsequent uncontrolled plastic pollution is one of the major environmental problems governments and agencies must face today.

The global production of plastic reached almost 370 million metric tons (Mt) in 2019 [36], almost 60 million of which are produced in Europe. The vast majority of the plastic products that enter the global market are durable materials, in particular, polypropylene and polyethylene are the leading polyolefins on the market, with the production of packaging being the main use of such plastics [36]. As of 2017, it was estimated that 8300 Mt of plastics were produced worldwide while, as of 2015, 79% of all plastic produced had been accumulating in landfills or the environment [37]. The UNEP reports that only 9% of all plastic ever made has been recycled, 12% has been incinerated and the rest accumulates in landfills or nature [38]. Today, 300 Mt of plastic waste are produced every year and around 80% of marine litter is due to plastic debris, with the infamous *“Great Pacific Garbage Patch”* being a dreadful testament to these numbers and with an estimate of 75,000 to 300,000 tons of microplastics entering EU habitats every year [38,39,40,41].

Plastic debris in the natural environment is extremely persistent, with degradation in seawater being estimated from hundreds to thousands of years [42,43]. Plastic marine debris results in severe, harmful, impact on the ecosystem [44]. Because of its long half-life and hydrophobic nature, plastic debris provides excellent conditions for the proliferation of diverse microbial communities, forming an ecosystem referred to as “plastisphere” [45]. The microbial action, together with mechanical stress, thermal and UV-light degradation, results in the fragmentation of the debris into microplastics, to the point that plastic residues can be found in many aquatic species, as well as birds and other wildlife [46]. In turn, this poses a risk to human health by entering the food chain [47,48,49].

A great part of the answer to plastic pollution comes from increasing recycling and repurposing of already produced plastics, as well as replacing several classes of plastic items, particularly single-use products, with recyclable alternatives, and from a change in mentality and habits in our society. At the same time, fossil resources are finite and their use results in greenhouse gas (GHG) emissions. As reported by the Ellen MacArthur Foundation in 2016 [50], it can be estimated that by 2050 the plastics sector *“will account for 20% of total oil consumption and 15% of the global annual carbon budget by 2050 (this is the budget that must be adhered to in order to achieve the internationally accepted goal to remain below a 2 °C increase in global warming)”* [50] (p. 7). The production of plastics from renewable sources has been suggested to achieve a lower carbon footprint, since the raw materials uptake carbon dioxide during their growth, and to alleviate the economy’s dependence on fossil fuel [13,41,50]. The application of biodegradable plastics in specific fields, such as soil cover films, carrier bags, and single-use packaging is also suggested as part of technological advancement in the bioeconomy [7,13].

### 1.2. Circular Economy and Bioplastics

Broadly speaking, a circular economy is an economic system and production model aimed at maximizing the reuse and recycling of resources, therefore extending the life cycle of products while minimizing waste. The model was thought of as a response to the traditional economy, the linear economy, where resources are used to manufacture products which are then used and discarded as waste. The *Circular Economy Action Plan* presented by the EC in 2020 [2] outlines the main directions towards which the economic model is being developed. We briefly summarize some of the main points made in the document.

Products should be designed with reusability and recyclability in mind, i.e., they need to be more durable, repairable, recyclable. Packaging is to be reduced, restricted to certain applications, and designed for recyclability. The production of single-use items is to be restricted and the destruction of unsold items is to be banned. Finally, more support to the bio-based sector is also considered as one way to enable greater circularity in industry, though it is also noted that the sourcing, labeling and use of bio-based, biodegradable and compostable plastics, are emerging challenges for which the EC will develop a policy framework [2] (p. 9). The topic of bioplastics is more extensively discussed in a 2018 EC action plan [51] focused on bioeconomy.

Overall, the EC communications suggest that the policy will be to support, e.g., via financial and regulatory incentives, the growth of the bioplastics industry, as one way to move towards a low-carbon economy [41,52,53]. For example, more than 100 million euros have been provided to finance R&D focused on alternative feedstocks, as part of the *Horizon 2020 Research Programme* [52]. The European Committee for Standardization (CEN) has also produced several harmonized standards in the past five years, covering methodologies to claim biodegradability and compostability, and to measure the bio-based content of plastics, to better regulate the bioplastic field. Still, it is acknowledged that more standards are required and that applications of biodegradable plastics can come with both positive and negative implications [53].

## 2. Bioplastics: Definitions and Market

The term bioplastic is often used loosely and synonymously to biodegradable. While some bioplastics are indeed biodegradable, not all are. Bioplastics should be intended as polymers that meet any of two criteria: the polymer is bio-based, the polymer is biodegradable [28,54]. Bio-based means that the polymer is either entirely or partially obtained from biomass, i.e., from any kind of organic renewable material of biological origin as well as organic waste. Biodegradable means that the material can break down into natural substances such as carbon dioxide, water and biomass, due to the action of microorganisms. In a more specific sense, a biodegradable plastic is a plastic material that complies with certain official standards of biodegradability, where a certain amount of degradation needs to be scientifically observed within a certain amount of time and under specific conditions. Similarly, a compostable plastic undergoes biodegradation in industrial composting facilities and has to comply with specific standards.

Bioplastics therefore form three broad groups of polymers: those that are both bio-based and biodegradable, those that are only bio-based and those that are only biodegradable. Some main examples of bioplastics that are both bio-based and biodegradable are polylactic acid (PLA) [55,56], polyhydroxyalkanoates (PHAs) [57] and bio-based polybutylene succinate (bio-PBS) [58], as well as plastics based on starch, cellulose, lignin and chitosan. Examples of bioplastics that are bio-based but not biodegradable are bio-based polyamides (bio-PP), polyethylene (bio-PE), polyethylene terephthalate (bio-PET) [59]. Finally, examples of biodegradable bioplastics that are based on fossil resources are PBS, polycaprolactone (PCL) [60], polyvinyl alcohol (PVA) [61] and polybutylene adipate terephthalate (PBAT) [62]. Furthermore, polymers like bio-PE, which are bio-based and chemically identical to their fossil-based counterparts, are typically referred to as drop-in polymers. Table 1 lists some bioplastics that are frequently encountered on the market or in research, classified on the basis of the origin of the raw materials and their biodegradability.

Today’s production volume of bioplastics is relatively small when compared to the numbers of the common plastic industry. According to European Bioplastics, the global production of bioplastics in 2018 was around 2 Mt [12], while the global production for plastics was around 360 Mt. At the same time it is anticipated that the global market for bioplastics will grow steadily for the next five year, increasing in volume by around 40% [12]. Different examples of bioplastics already exist on the market and are produced by companies both in Europe, the USA and Asia, with some of the most important manufacturer being BASF (Germany), Corbion N.V. (Netherlands), NatureWorks LLC (USA), CJ CheilJedang (Korea), Novamont (Italy), Tianjin Guoyun (China). Two historically successful examples are Cellophane^TM^, produced from regenerated cellulose by Futamura Chemical Company (UK), and Nylon-11 produced from castor oil by different manufacturers. More examples are the PLA branded Ingeo^TM^ produced by NatureWorks LLC, as well as the Luminy^®^ series of PLA resins produced by Total Corbion (fifty-fifty joint venture between Total and Corbion), which is also working on the production of bio-based PEF; Corbion distributes its PURASORB^®^ grades of bioresorbable polymers, which include PLA, PCL and copolymers; Danimer Scientific produces the PHA-based bioplastic Nodax^TM^; several compostable polymers are produced by BASF (ecoflex^®^, ecovio^®^); Novamont produces its biodegradable, starch-based, Mater-Bi^TM^; Arkema produces a series of bio-PA (Nylon) under the name Rilsan^®^.

### 2.1. Production Routes of Bio-Based Plastics and Main Examples

As already introduced, bio-based plastics are entirely or partially obtained from some type of biological source, this includes plants, microorganisms, algae, as well as food waste. Some bio-based plastics are obtained from polymers that form directly in nature, within microorganisms and plants. Notably, cellulose—the most abundant organic compound and the main constituent in plant fibers—has been used ever since the 19th century. Other bio-based plastics are relatively novel and are obtained through synthetic routes making use of natural resources to formulate monomers which are then polymerized. In general, we can identify three main routes to produce bio-based plastics: (1) polymerization of bio-based monomers; (2) modification of naturally occurring polymers; (3) extraction of polymers from microorganisms. Table 2 lists some of the main bio-based polymers grouped by their production route and followed by a brief description of their synthesis.

Today, several bio-based polymers are produced through the polymerization of monomers obtained from natural sources, PLA being the primary example. Polylactic acid is a thermoplastic aliphatic polyester obtained from the fermentation of plant-derived carbohydrates, e.g., sugars obtained from sugarcane or sugar beet, or starch obtained from corn or potato. The fermentation process makes use of various microorganisms, typically *Lactobacilli* strains, which convert sugars to lactic acid [55,63]. If starch is used as feedstock, this is first enzymatically converted to sugars (glucose). Most commonly, the lactic acid is then polymerized to low molecular weight (Mn) PLA oligomers, which are in turn depolymerized to yield lactide, the cyclic dimer of PLA. The ring-opening polymerization (ROP) of lactide will then yield high Mn PLA [55,64,65]. Due to the chiral nature of the monomers, L(-) and D(+), in use during the polymerization process, three stereochemical forms of PLA can be obtained. Depending on the ratio of L- to D-isomers, the resulting polymer can be amorphous or show different degree of crystallinity, with influence on degradation [66] and mechanical properties [67]. PLA processability is comparable to many commodity thermoplastics, which leads to its use as packaging material [56,68]. PLA is also recognized as biodegradable and compostable [55,64,65], it is therefore used in the production of compost bags and disposable tableware, and other applications where recovery of the used product is not feasible. Furthermore, PLA biocompatibility has made it into one of the most important polymers in biomedicine and tissue engineering [55,64,65,69]. Finally, PLA is one of the main materials in use to produce filaments for fused deposition modeling, a common 3D printing manufacturing process [70].

PBS is another thermoplastic polyester that can be produced from the microbial fermentation of sugars derived from natural feedstocks. The typical route of production for PBS is the esterification of succinic acid with 1,4-butanediol [71], where the succinic acid can be obtained from the anaerobic fermentation of bacteria or yeast and subsequently reduced to 1,4-butanediol. Several microorganisms have been studied for the biosynthesis of succinic acid, e.g., *Anaerobiospirillum succiniciproducens* and *Actinobacillus succinogenes* [72]. The polymerization process proceeds through a first step during which the 1,4-butanediol is reacted with the succinic acid to yield oligomers of PBS, and a second step of polycondensation of the oligomers to yield semicrystalline, high M_n_ PBS [58]. PBS shows similar properties to polyethylene terephthalate and polypropylene and finds applications as compostable packaging and bags, as mulch film and hygiene products [58,73]. The use of PBS in biomedical applications has also been attracting significant attention, thanks to its biodegradability and low toxicity profile, though its low flexibility and slow degradability rate need to be circumvented by blending or copolymerization with other polymers, such as PLA [71,73].

Bio-based polyethylene is an aliphatic thermoplastic synthesized from the polymerization of bioethanol. The bioethanol is obtained through the fermentation of sugars from the aforementioned feedstocks (sugarcane, sugar beet, and starch from corn, wheat or potato) [59], yeast or bacteria being used as fermentation agents [74]. The bioethanol is distilled and dehydrated to obtain ethylene which is then polymerized to bio-PE. The polymer is equivalent to fossil-derived polyethylene and the same different types (low and high density, linear and branched) can be obtained, consequently, bio-PE can be used for any of the many applications of PE. It should also be noted that bioethanol can also be used in the synthesis of other important plastics such as polyvinyl chloride, polystyrene and polyethylene terephthalate [59].

Several naturally occurring polymers can be used to produce bio-based and biodegradable plastics, in particular the polysaccharides starch and cellulose.

Among naturally occurring polymers, cellulose is the most abundant one, being ubiquitous in plants. It is a structural polysaccharide based on repeating units of D-glucose. Cellulose has attracted great attention from research and industry due to its abundance, low-cost, biocompatibility and biodegradability. Cellulose is typically obtained from wood through a pulping process and can be converted to different materials, in particular two main cellulose-based plastics (or cellulosics) are regenerated cellulose and cellulose diacetates [75]. In the production of cellulose diacetates, the cellulose is first converted to cellulose triacetate by reaction with acetic anhydride, this is then partially hydrolyzed to obtain a lower degree of substitution. Most typically cellulose diacetates are produced with degree of substitution around 2.5. Cellulose acetates find several applications in the textile industry [76], as fibers in cigarette filters [77], films (e.g., photography) and membranes in separation technologies (e.g., hemodialysis) [78]; manufactured as porous beads they have potential applications in biomedicine and biotechnology [79]. Cellulose diacetate is also biodegradable under different natural conditions with the process being accelerated by hydrolysis [80].

Regenerated cellulose is typically prepared following the viscose process (though other industrial methods exist), in which cellulose is converted to cellulose xanthogenate by reaction with alkali and carbon disulfide. The intermediate is dissolved in NaOH solutions, resulting in a mixture called viscose, which can be processed as films and fibers and treated in acidic solutions to yield regenerated cellulose [81,82]. Regenerated cellulose materials are either already applied or could find applications, in different fields, from textile and packaging, to biotechnology and biomedicine [82]. Rayon and cellophane, which are generic trademarks for regenerated cellulose fibers and films respectively, are materials with great commercial importance. Rayon finds many applications in the textile industry, from the manufacture of clothing to the production of wound dressings [83]. Cellophane is almost ubiquitous in the food packaging market, but also in the cosmetic (casing, boxes, etc.) and pharmaceutical industry [82].

Starch-based polymers form an important family of bioplastics on the market. Starch is a polysaccharide consisting of two main macromolecules, amylose and amylopectin, and is obtained from feedstocks such as corn, rice, wheat or potato [84,85]. Thermoplastic starch (TPS) is the material obtained from a granular form of native starch, through thermomechanical processing (extrusion) with the addition of gelatinization agents or plasticizers [84,85,86,87]. Typical plasticizers in use to improve the processability of TPS are glycerol and other polyols, sugars, amides and amines, and citric acid [84]. TPS can be used on its own, though very often it is used as part of polymeric blends with polymers such as PLA and other polyesters, to improve its properties. Starch-based plastics find different applications in the packaging, food, textile and pharmaceutical industry [88,89,90].

Bacteria can synthesize and accumulate a large number of biopolymers, many of which can be potentially exploited for industrial applications or as high-value products in the medical field [91]. Polyhydroxyalkanoates are a family of polyesters synthesized by the activity of several types of bacteria, where they accumulate serving the purpose of carbon reserve material. The intracellular accumulation of PHAs is typically promoted by particular culturing conditions and nutrients starvation, which can lead to high concentration of accumulated polymer [71,91,92,93,94]. Several renewable feedstocks, as well as carbon dioxide, chemicals and fossil resources, can be used as substrate for the production of PHAs [94]. In a typical process a seed culture containing the chosen bacteria is inoculated in a fermentation vessel containing the fermentation medium. At the end of the culturing period, the polymers can be obtained by solvent extraction, separated from the residual biomass and reprecipitated by mixing with a non-solvent, typically an alcohol [31,71,95]. To this day, more than 150 monomeric units have been identified, which can lead to different polymers with different properties. Polyhydroxybutyrate (PHB) is the simplest PHA and the first one to be discovered in the bacterium *Bacillus megaterium*. PHAs find applications in the packaging, food and chemical industry, though most recently attention has been shifting towards possible agricultural and medical applications [96,97,98].

### 2.2. Biodegradability and Compostability Standards

As introduced, biodegradable polymers are susceptible to be broken down into simple compounds because of microbial action. Many plastics have been known to undergo this process in a reasonably short time (e.g., six months), and are commonly identified as biodegradable, though to substantiate biodegradability claims, certain standards have been put into place in the past twenty years. These standards present methodologies to evaluate the biodegradability and compostability of a plastic, where compostable refers to the material being degraded under specifically designed conditions and by specific microorganisms, typically in industrial composting facilities. The main standardization bodies involved are the International Organization for Standardization (ISO), European Committee for Standardisation (CEN) and the American Society for Testing and Materials (ASTM). Table 3 reports the main ISO [99] and CEN [100] standards in place, many of which are shared as the CEN standards are often based on the ISO ones. In particular, for a polymer to be marketed as biodegradable or compostable the main standards to conform to are the European EN 13432 or EN 14995 or the international ISO 17088 (other equivalents would be the USA ASTM 6400 or the Australian AS4736). As part of the requirements to pass the standards, the testing methodologies in use to evaluate biodegradability need to be the ones outlined by other official standards, for example EN ISO 14855. The simulated environment, the biodegradability indicator in use, the inoculum in use, test duration, number of replicates required and percentage of evaluated biodegradability to pass the test, are focus points for biodegradability testing standards. It can be noticed that the biodegradability evaluation is carried out by different experimental methodologies, such as release of carbon dioxide and oxygen demand measurements. Indeed, the main indicators of biodegradation adopted by these standards are the measurement of BOD (the biological oxygen demand) or the measurement of evolved CO_2_, though also mass loss measurements, measurements of CH_4_ evolution, as well as surface morphology and spectroscopy analysis are methodologies in use [101]. Biodegradability standards describe a series of well-defined conditions under which biodegradability or compostability tests are to be carried out, for example temperature, microbial activity and humidity. While this is required for reproducibility and repeatability of results, researchers have pointed out the difficulty in encompassing the variability of conditions encountered in natural, open environments [10,14]. In particular, the more environmentally harmful perspective of plastic waste leaking into the natural environment leads to a series of possible environmental conditions that are hard to accurately predict and simulate. For example, plastic debris leaked in the sea is exposed to a wide range of temperatures, depending on climate, biomes, buoyancy, and other characteristics that might very well change over time. Given that the appeal of biodegradable plastics in several applications is their supposed ability to degrade in the environment, completely and harmlessly, it is of utmost importance to understand the validity of these standards outside of laboratory conditions. In a 2018 review of biodegradability standards, Harrison et al. [35] found that the international standards in use would be insufficient to predict the biodegradability of carrier bags in aqueous environments (wastewater, marine and inland waters). They concluded that the standards in use would typically underestimate the time required for polymers to undergo biodegradation in a natural, uncontrolled environment, particularly because of the methods in use relying on artificially modified media and inocula and relatively high temperatures, that do not reflect what is commonly expected in a natural environment. In 2017, Briassoulis et al. [102] come to similar conclusions when reviewing the standards relevant to the biodegradability of plastics in soil, particularly for the agriculture and horticulture environment. They observe that the standard methodologies enhance the conditions for biodegradation of the specimens in a way that may not be representative of the natural environment, where temperature, water content and soil properties can vary considerably. The standards caution the users about the potential difference between laboratory and natural environment results, though it is not clear how the methodologies should be modified to obtain more representative conclusions. In a 2017 work, Emadian et al. [103] reviewed a series of studies on the biodegradation of bioplastics in different conditions. For the same biopolymers, they reported extremely different results across the studies taken into consideration. For example, testing the biodegradability of PLA in compost researchers reported biodegradation values as low as 13% over 60 days [104] and as high as 70% over 28 days [105]. This difference in results is due to different methodologies, conditions and sample geometry and size being in use, and therefore stresses the need for standard procedures being followed during laboratory tests. Though, at the same time, the kind of differences that create this discrepancy, can also be encountered in a natural environment, and further stress the problem of how well biodegradability can be predicted outside of a laboratory. Further problematics can be encountered when considering the biodegradation of polymeric blends instead of homopolymers. In a 2018 paper Narancic et al. [14] reported on the biodegradability of several biopolymers and their blends in different environments, following the relative standards. The paper is extremely thorough, and its full analysis goes beyond the scope of this review, though one conclusion was that the polymeric blends would generally biodegrade well under industrial composting conditions, but they would show poor biodegradation in aquatic environment and soil. Interestingly, the authors observed that, while PLA is generally not home-compostable, when blended with PCL it would result in a material that could undergo biodegradation under home-composting conditions (though not in soil). At the same time, this was not the case for blends of PLA and PHB, which remained not home-compostable.

**Figure 1:**
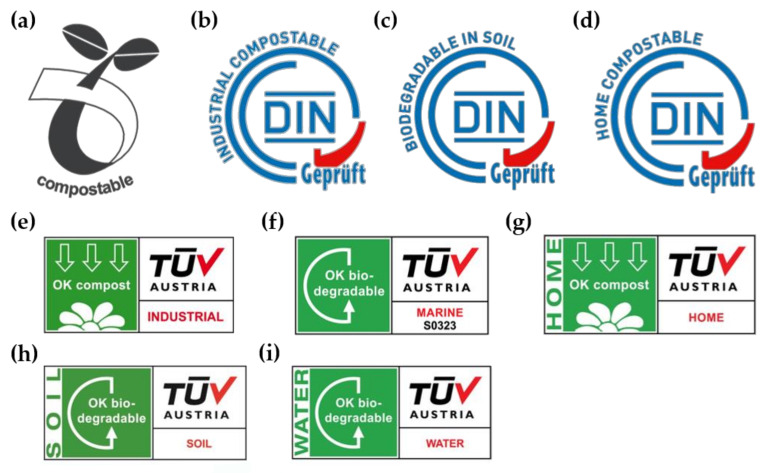
Certification labels relating to biodegradability and compostability: (**a**) seedling logo by *European Bioplastics*, indicates that the product is industrially compostable and complies with EN 13432; (**b**–**d**) DIN CERTCO labels for industrial compostability, biodegradability in soil and home compostability, respectively; (**e**–**i**) TÜV Austria labels for industrial compostability, marine biodegradability, home compostability, soil biodegradability and freshwater biodegradability, respectively.

Several companies in Europe market their products with labels specifying their biodegradability. Figure 1 summarizes the main certifications in use in Europe, which are released by the Belgian certifier TÜV Austria and German certifier DIN CERTCO. It should be noted that home compostability is yet to be specifically described by EN harmonized standards, though standard prEN 17427:2020 “Packaging-Requirements and test scheme for carrier bags suitable for treatment in well-managed home composting installations” is pending. Industrial compostability is covered by the standards EN 14995 and EN 13432, and these are used during the certification. Soil biodegradability is also certified by two of the labels through EN 17033, which therefore limits the certification to mulch film. Two labels for biodegradability in water are offered by TÜV but they are independent from EN standards.

### 2.3. Overview of Abiotic and Biotic Degradation Mechanisms

While the official definition of biodegradation is exclusively focused on the biotic phenomena, it is important to remember that abiotic phenomena take place during the biodegradation of a polymeric material, and these can have a strong influence on the overall degradation rate. We can identify three main steps through which biodegradation proceeds, with the process being susceptible to stop at each step [103,106,107,108].

During a first step referred to as biodeterioration, the material is broken down into smaller fractions due to biotic and abiotic activity. During this step a biofilm is formed on the surface of the material, consisting of a variety of microorganisms embedded in a matrix of water, proteins and polysaccharides produced by the same microorganisms [109,110]. The process of colonization of a polymeric surface by a microbial biofilm is referred to as fouling and follows different steps that lead to the settlement of bacteria and other microorganisms (microfouling) as well as larger organisms (macrofouling) such as larvae [110,111]. During and subsequently the biofilm formation, the microorganisms can infiltrate the surface porosity of the polymer which results in a change of the porous volume and potentially in cracks, furthermore this process facilitates water infiltration and consequentially hydrolysis. Additives and plasticizers can also leach out of the polymer during this step, resulting in embrittlement and rupture.

The microorganisms inhabiting the biofilm secrete enzymes that can be broadly defined as intracellular and extracellular depolymerase [28,101,103,106,110]. These enzymes are responsible for the second step in biodegradation, the depolymerization step, during which the polymer chains are broken down into shorter oligomers and eventually monomers, though this process can also result from abiotic phenomena which are covered later in this section.

The third step of biodegradation comprises of the assimilation and mineralization processes during which monomers and oligomers from the broken-down polymer can reach the cytoplasm and enter the metabolism of the microorganisms, therefore being converted to metabolites, energy and biomass, with the release in the environment gases, organic compounds and salts [106]. This step is of particular importance given that several standardized methodologies rely on the analysis of evolved CO_2_ to evaluate biodegradability.

Abiotic degradation phenomena are involved either before or in concomitance with biotic degradation. Typical abiotic degradation phenomena are mechanical, thermal, UV, and chemical degradation.

Mechanical damage, both at macro and microscopical scale, can facilitate and accelerate other types of abiotic and biotic degradation, for example by increasing the available contact surface or creating defects that are easily attacked by chemical infiltration and more susceptible to heat damage.

Heat can further increase mechanical damage by lowering the mechanical properties of the polymer, e.g., if the plastic were to experience temperatures higher than its glass transition or melting temperature, its structural integrity would be quickly compromised under relatively low forces. Conversely, temperatures much lower than the glass transition might result in brittleness and rupture of the polymer. The loss of crystallinity, as well as the transition to the rubbery state, can also increase the permeability of biotic and abiotic agents in the polymeric matrix, therefore accelerating the degradation process. This is particularly important for polyesters, such as PLA, where the degradation process is strongly governed by hydrolysis reactions and therefore will proceed at a much faster rate when water can easily penetrate the polymeric network.

Chemical degradation includes oxidative phenomena due to molecular oxygen and is, therefore, one of the main factors in abiotic degradation. Oxidation often proceeds concomitantly with light degradation phenomena, leading to the formation of free radicals, ultimately decreasing the molecular weight by chain scission as well as causing crosslinking of the polymeric network which often leads to high brittleness. Hydrolysis is the other main factor acting during chemical degradation. Several bioplastics contain hydrolyzable covalent bonds, e.g., ester, ether, carbamide groups. Chemical degradation acts synergistically with all other degradation mechanisms. For example, oxidation and hydrolysis are facilitated by the polymer transitioning to the rubbery state and additionally losing its crystallinity due to exposure to relatively high temperatures.

UV-light degradation, or photodegradation, is also a very common occurrence in everyday life plastics. Photodegradation can typically lead to radicalization, resulting in chain scission and/or crosslinking, as already discussed these phenomena can be concomitant to oxidative degradation. Typically, photodegradation will result in the plastic material break down, which in turn increases the surface area available for biotic degradation to occur, and ultimately speeding up the biodegradation process. It can therefore be expected a large difference in biodegradation times depending on the plastic debris being exposed to sunlight or less; this could be the difference between a plastic bag floating at the sea surface against dense plastic debris sinking to deep-sea level.

## 3. Life Cycle Assessment of Bioplastics

The topic of bioplastics sustainability is very much debated in our society, both at the academic and institutional level. Life cycle assessment (LCA) is the main approach through which researchers and policymakers can investigate the benefits and drawbacks of using bioplastics in place of common plastics. LCA is a standardized methodology, with the prevalent standards being ISO 14,040 and ISO 14044, through which it is possible to analyze the environmental and socio-economic impacts related to the production and use of a certain good. There exist several LCA standards in use at the international level, as well as guidelines that are valid in the EU. The *Product Environmental Footprint* (PEF) and the *Organisation Environmental Footprint* (OEF) [112] are two methodologies compiled by the Joint Research Centre (JRC), the department of the EC focusing on scientific research. The JRC also authored the technical report *Comparative Life Cycle Assessment (LCA) of Alternative Feedstock for Plastics Production* [113] which builds on the PEF providing scientific guidelines and modeling methodologies for an audience already expert in LCA. These guidelines are themselves based on ISO 14040 and ISO 14044. The two standards describe the various aspects of performing an LCA, from definitions and goals to its main phases of life cycle inventory analysis (LCI) and life cycle impact assessment (LCIA), respectively, the “data collection and calculation procedures to quantify relevant inputs and outputs of a product system” and the process through which the inventory data is associated with specific environmental impact categories [114]. Table 4 reports some of the main ISO and CEN standards relevant to the life cycle assessment of plastics and bioplastics, some of the standards are shared as the EN version is based on the ISO one. Among these standards, ISO 14040 and ISO 14044 are the main ones that define the principles and practices for LCA, providing the basic framework for the assessment but leaving a range of choices to the practitioners. Further guidance is provided by the International Reference Life Cycle Data System (ILCD) which consists of technical documents and a data network aimed at ensuring the validity and consistency of LCA. General definitions and aspects of bio-based plastics are also discussed by the CEN standard EN 17228, as well as in EN 16575 which represents a vocabulary for bio-based products.

The complete LCA of bioplastics needs to take into consideration several impact categories, of environmental, social and economic nature, as well as different options for the product EOL. Typically, this is not the case with most LCA research, as data on the entirety of a certain product’s life might be lacking or because of the intended motivation of the study not requiring assessing the whole life cycle. Depending on the system boundaries, we can identify two main approaches to the LCA of a product: the cradle-to-gate and the cradle-to-grave assessment [113,115,116]. Cradle-to-gate refers to an assessment from the resource extraction stage (cradle) to the factory gate, meaning at the end of production. For bio-based products this includes crops cultivation and biomass pre-processing, and in general, any transportation involved should be included. Additionally, eco-profile is a name in use to describe the cradle to factory gate life cycle inventory assessment of polymers and chemicals. Eco-profiles are used as building blocks in cradle-to-gate LCA studies and many can be found available on PlasticsEurope website [117] as well as on other databases (Sphera, GaBi, openLCA Nexus, SimaPro Industrial Database, Life Cycle Initiative).

The cradle-to-grave assessment takes into consideration the entire life cycle of the product, from the raw material extraction to the EOL management. This includes all aspects taken into consideration by the cradle-to-gate assessment and also the product’s retail, storage, its use by consumers and its disposal.

LCA needs to consider several impact categories and put them into comparable numbers depicting the potential influence on the environment, these categories are therefore standardized in their definitions and units in use [113,115,118,119]. Table 5 reports the main indicators in use for the different impact categories [118]. The global warming potential (GWP_100_) is the main parameter reported by LCA academic studies, it gives an indication of the amount of GHGs produced by the system under assessment and the effect on global warming. Several GHGs are released during the production and distribution of a certain good, each having a different potential for global warming, which is defined by the specific amount of infrared radiation the gas can absorb in the atmosphere. The GWP_100_ considers the overall global warming potential by converting each mass of gas emitted to the atmosphere, in the mass of equivalent CO_2_ that would absorb the same amount of energy. Furthermore, the GWP depends on the number of years over which the energy absorption is calculated. Typically, 100 years are considered, hence the subscript in GWP_100_.

LCA studies typically take into consideration environmental impact categories, though also social and economic aspects are of great importance. Social life cycle assessment (S-LCA) looks at how the extraction or production of raw materials, and manufacturing, distribution, use and disposal of goods, can bring negative effects from a social point of view [20]. Life cycle costing (LCC) is also referred to as environmental LCC (E-LCC) [20] when applied in conjunction with LCA. E-LCC takes into account all costs that are involved with the product’s life cycle, independently from the party incurring such costs. Environment-relate cost factors are taken into consideration, such as ecological taxes and expenses for emissions control.

### 3.1. Life Cycle Assessment Research on Bioplastics

LCA research has already been reviewed in the past years, here we discuss some of the main findings.

In 2018 Spierling et al. [20] presented a review of cradle-to-gate LCA, S-LCA and LCC studies on different bioplastics, and focused their attention not only on the environmental impacts but also on the social and economic impact assessments. Assessing CO_2_ emissions related to bio-sourced plastics, the authors underline that since bioplastics are often considered carbon-neutral, the data about carbon content and CO_2_ uptake are often omitted. This lack of information leads to an imbalance in modeling, especially if the EOL stage is such that methane or GHGs other than CO_2_ are released. The authors observed that many LCA studies did not provide information on important impact categories, such as acidification, while mostly focusing on GWP and energy depletion. Land use is also reported by different studies, but other than reporting the square meters occupied no related impacts are considered. Overall, the authors could only compare the LCA studies on the GWP basis and estimate potential savings of 241–316 million tons of CO_2_-eq per year by replacing 65.8% of conventional plastics with bio-based ones. Still, the authors acknowledged several limitations and uncertainties and noted that the assessment of the use phase and EOL phase could strongly impact the results. Regarding S-LCA and LCC, the authors report the lack of studies and available data, though the authors could infer a high social risk potential when bioplastics’ raw materials are produced in countries with weak legal standards.

In a 2020 review, Walker and Rothman [33] assess the comparative LCA of bio-based and fossil-based plastics, focusing on environmental LCA, cradle-to-grave, studies. The reviewed studies were checked for compliance with the EU PEF, though none of the studies completely met the document requirements. Partially complying papers were therefore selected; seven bio-based polymers and seven fossil-based polymers were compared across seven impact categories for which sufficient data were available. The authors report a lack of agreement between different studies, both for bio-based and fossil-based polymer assessments, with variations as high as 400% for the same impact category and same polymer. Negative values of CO_2_ emissions were noted for bio-based systems due to the gas being absorbed during biomass growth. Like in Spierling et al., the authors note that this a controversial point as potential CO_2_ or methane emissions during EOL are often not considered. Indeed, the authors underline that in cradle-to-grave studies several impact categories show far worse values than the ones in cradle-to-gate studies, which is due to the emissions and energy consumption during several EOL options. Furthermore, the authors notice that many studies on biodegradable polymers assumed composting as the EOL phase, though assuming incineration would significantly increase particulate emissions. Generally, the authors report similar values for most impact categories across fossil-based and bio-based systems. Finally, they conclude by commenting on the lack of comparability and standard methodologies in the field, stating that “without the ability to compare across studies, LCA has much lower relevance than it could or should have”.

In a recent review, Bishop et al. [34] also compared results from bio-based and fossil-based polymers LCA studies. Similar to the other works discussed, the authors found a lack of impact categories being covered as well as a lack of uncertainty analysis being carried out. Additionally, they noted that most studies did not account for the use of additives and their potential leakage in the environment and suggested that LCI should always include additives in use even if used in small quantities. In line with the other review already discussed, Bishop et al. note that the assumption of bio-based plastics being carbon-neutral can be misleading. In addition to the previous studies, they observe that biogenic CO_2_ will spend a period of time in the atmosphere depending on the growth cycles of the biomass. While this might be negligible for fast-growing crops, it can become relevant with an increasing use of lignocellulosic-derived biomass which has long growth cycles. The authors underline that the worst approach to the assessment of bioplastics is to imply a large and permanent CO_2_ uptake since most certainly this amount of gas will be released once again to the environment in the 100-year time horizon for which the GWP is typically calculated.

An important issue for bioplastics is their durability in service conditions, i.e., the ability of biopolymers to maintain unchanged the properties and performance in service. To be ensured unchanged properties, the biopolymers are usually added with appropriate stabilizing systems, such as synthetic antioxidants, UV-absorbers, quenchers [120] or naturally occurring molecules having protection actions [121,122,123]. All stabilizers can protect the biopolymers and improve the oxidative resistance in service conditions, making these materials suitable and durable. Interestingly, as documented, some natural antioxidants can exert concentration dependent anti-/pro- oxidant activity and, if they added at high concentrations, can exert pro-oxidant activity rather than protection action [124,125,126].

From what already discussed, one very important, though unfortunately overlooked, aspect of LCA is the assessment of the EOL phase. In the following section we report on research focused on the EOL of bioplastics.

### 3.2. End-of-Life Options for Bioplastics

The analysis of the EOL of a product is one of the most important parts of LCA, here we report some of the main research works focused on the topic.

In a 2013 review, Soroudi and Jakubowicz investigated the mechanical recycling of bioplastics and their blends [127]. The authors concluded that the performance and sustainability of recycled bioplastics could not be thoroughly understood given that research in the field was at a preliminary stage. They noticed that additives, such as compatibilizers, would need to be considered for the mechanical recycling of blends of bioplastics, because of the immiscibility of polymeric mixtures. They also noticed that biocomposites’ mechanical properties often depend on the microstructure obtained during processing, for example, alignment of fiber fillers, which can be lost after recycling resulting in the loss of mechanical performance. They observe that chemical recycling, as an alternative to mechanical recycling, can be very costly, for example, the depolymerization of PLA requires high temperatures and therefore high energy expenditure. The authors observed that mechanical recycling of plastics with bioplastics might result in increased contamination, therefore affecting the performance of the recycled material.

In a 2016 paper, Cosate de Andrade et al. [128] presented an LCA of PLA comparing chemical recycling, mechanical recycling, and composting. The authors found that mechanical recycling had the least environmental impact, followed by chemical recycling and lastly composting when considering the climate change, human toxicity, and fossil depletion categories. In particular, it was observed that composting was outperformed as EOL option since it does not produce PLA or starting blocks for PLA, and therefore it will cause all the impact associated with producing virgin polymer again.

In a 2017 paper, Hottle et al. [24] investigate the EOL phase of several biopolymers and fossil-based polymers. The three EOL options taken into consideration are recycling, composting, and landfilling. As an important note, the paper takes into consideration the waste collection and transport stages. The two stages are required independently from the EOL option and can strongly influence the overall impact. The authors observed that the EOL stage greatly influences the overall sustainability assessment of a polymer life cycle. Recycling was found to be the most effective way to reduce environmental impacts for the drop-in plastics under assessment. Large, negative, values of GWP, ADP-fossil, and other impact categories are achieved through recycling of these polymers since the original raw material is bio-based (bioethanol) and because it is assumed that the recycled material offsets the fossil-based production of virgin polymers. A worst-case scenario for compostable polymers being landfilled is the uncontrolled release of methane following their biodegradation. In this case, the authors found that the GWP would drastically increase, particularly for PLA. On the other hand, composting would drastically reduce ODP, GWP, as well as eutrophication. Transportation, including international shipping, waste collection, intermediate handling, all largely contributed to higher GWP and ODP. Port-to-port shipping from California to recycling facilities in Hong Kong was considered and resulted in large increases in several impact categories.

In a 2020 review, Lamberti et al. [129] cover the recycling routes of several bio-based polymers. The authors observe that, generally, the best practice is to reuse any plastic as much as possible before recycling, then the plastic should be mechanically recycled until the resulting material is of commercially acceptable grade, finally, the plastic should be chemically recycled to recover part of the original monomers. For example, they observe that mechanical recycling of PLA results in a lower grade polymer that can be reused, though further recycling should be chemical. The authors report that chemical recycling of PLA through alcoholysis generates value-added products (different lactates depending on the alcohol), which can also be converted to lactide which can be directly polymerized to high M_n_ PLA. Furthermore, bio-PET and bio-PE can be mechanically recycled multiple times before being chemically recycled. They identified glycolysis as the best chemical recycling option for bio-PET and pyrolysis as the one for bio-PE. The former results in value-added chemicals and in the least number of steps to polymerize back to PET, while the latter is the only route able to degrade PE resulting in valuable aromatics and fuel (gas and char). The authors conclude that biopolymers’ mechanical performance needs to be improved and that better schemes for recycling and waste collection need to be put into place.

Anaerobic digestion is an appealing waste management route for compostable bioplastics. The process converts municipal organic waste to biogas (methane) and can incur the problem of plastic bags contamination. The use of bioplastic bags for municipal organic waste could therefore prove to solve the contamination problems while converting unavoidable plastic waste into energy.

In a 2018 review, Batori et al. [130] reported on the conditions needed for the effective anaerobic degradation of bioplastics. They noted that typical biogas plants operate with a solid retention time of 15 to 30 days and, therefore, bioplastic bags should be able to degrade in this time length. The authors found PBS to be not suitable for the application because the polymer does not degrade under the conditions in use at the plants, they reported that PLA, PCL and PVA do not sufficiently degrade in the time range, but they found PHB to be suitable. It was observed that more standardization for biodegradable bags suitable for organic waste collection is needed, and the authors suggest that the standards should require biodegradation greater than 50% over 1 month. It was pointed out that the plastic bags should also be able to withstand moisture until they reach the fermenter, a property that might not be common in many biodegradable plastics due to the presence of hydrophilic moieties. The authors observe that less resistant bioplastics might be still used and coated in a layer of water-resistant ones.

In the previously referenced work, Narancic et al. [14] tested several bioplastics and blends, observing that the majority would degrade by thermophilic anaerobic digestion with high biogas output. Still, the authors reported that the degradation times were 3 to 6 times longer than the retention times of commercial plants.

In 2018 Zhang et al. [131] reported on the anaerobic biodegradation of 9 bioplastics compliant with standard EN 13432. The plastics, together with organic waste, were fed daily to a digester for 177 days with a retention time of 50 days. The authors found that the digestion process was not inhibited but they also observed that only 4 cellulose-based materials showed substantial biodegradation.

Researchers have also taken into consideration the result of biodegradable plastics entering the recycling process of common plastics. Different research groups investigated the effect of small (5 wt%) amounts of PLA being mixed in the recycling process of PET [132,133,134,135,136]. The results showed that even small quantities of PLA will negatively affect the mechanical and thermal properties of recycled PET, which can cause technological and economic burdens. The main problems are due to the difference in thermal degradation temperatures, with PLA already degrading at the processing temperature of PET and causing yellowing of the product. The polymers are also immiscible, which can cause a lack of homogeneous surfaces, undesired opaqueness, and defects or failure during injection molding.

Currently published study deals about that the oxidation during burning of biopolymers and synthetic polymers can produce similar amount of CO_2_. Therefore, a correct waste collection and management is required to solve the environmental troubles arising from the uncontrolled plastic use, release and accumulation of both petroleum-based polymers and bio-based polymers. The sustainable live vs. waste management of polymers and biopolymers depends on accurate end-of-life disposal of these materials and reduction of volumes of used plastics and bioplastics, maybe just articles having short service-life [137].

## 4. Bioplastics: Summary of Opportunities and Possible Challenges

Table 6 presents a summary of possible advantages and disadvantages related to the adoption of bioplastics, as discussed in the available literature.

While it should be kept in mind that the volume of bioplastics in our economy is still too small to accurately predict all implications resulting from large-scale adoption, one point in favor of their adoption is that fossil resources are limited. The adoption of bio-based plastics can, therefore, prove to be an important way to decouple our economy from an unsustainable model.

Biodegradable plastics might also prove to be beneficial in mitigating some environmental risk scenarios where the leakage of plastics is not easily avoided, nor the use of plastic items can be effectively discontinued. For example, mulch films need to be collected and recycled at the end of their life-cycle. However, they are contaminated with soil and organic material which makes recycling procedures economically unviable. Furthermore, film fragments can accumulate in the soil, causing an environmental risk. Biodegradable mulch films have been on the market since the early 2000s, offering the same performance as common plastic ones, while being biodegradable in soil [138].

Compostable plastic bags for organic waste collection are already in use, which eliminates the need to separate the bag from the waste. Their use in anaerobic digestion facilities–if the material is designed to degrade within the retention time–can also lead to the production of renewable energy.

**Figure 2:**
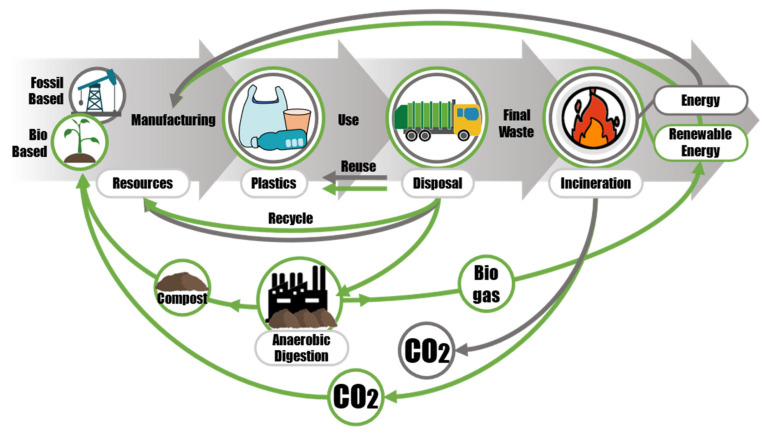
A simplified infographic representing the main steps in linear economy (straight arrows) and the additional steps introduced by circular economy with a focus on bioplastics (green arrows), considering anaerobic digestion as EOL option for compostable plastics, reuse and recycling for durable bio-based plastics and incineration as final disposal of any plastic that is no longer recyclable nor reusable.

Figure 2 presents a simplified infographic representing the main steps in plastics linear economy and what the authors consider some important additional steps introduced by bioplastics in the circular economy. The linear economy route proceeds through the collection of resources, the production of plastic goods, their use, and their disposal. The circular economy route adds two important steps of repurposing of goods and recycling (mainly mechanical) to extend the life of the material as much as possible. Durable bio-based plastics, like drop-in plastics, can be recycled and their goods can be repurposed like common plastics. Compostable plastics would be used primarily as food waste collection bags and contribute to the production of biogas and compost in appropriate industrial facilities. The compost would be used for agricultural purposes, including growing the raw materials for bioplastic production, and the biogas would provide energy, including energy for manufacturing processes. Bioplastic goods that are no longer recyclable would be incinerated to produce energy.

The bioplastic industry is still very small in volume and relatively new, when compared to the common plastic industry, furthermore regulations about bioplastics have been changing in recent years. Therefore, it is understandable that several present and future challenges related to the adoption of bioplastics can be identified. In the following sections we review the challenges that can be identified from what discussed so far.

### 4.1. Lack of Comparable LCA Studies

The use of different approaches and methodologies, as well as different reference units and data sources, strongly hinders the comparability of LCA studies. As already discussed, the use and EOL phases are often not taken into consideration, most assessments being cradle-to-gate. Yet, the EOL phase is shown to drastically influence the overall values of most impact categories. Studies that do not consider this stage can also come to the misleading conclusion that the production of a certain bio-based plastic completely removes GHGs from the atmosphere by converting CO_2_ to biogenic carbon, while, depending on the EOL, stronger GHGs might be produced. The use of the same reference units is also important to compare different studies. LCA studies of specific products should consider a reference number of items produced. This approach is more comparable across different studies than using mass as reference, because not all polymers are converted to the same number of items per unit of mass.

Moving forward, studies should be thought-out to be comparable so that the overall significance of LCA could be ensured.

### 4.2. Issues Relating to Standards and Regulations

In the last ten years, a lot of efforts have gone into the production of standards to define and evaluate compostability and biodegradability. While standards on industrial composting can reproduce the designed conditions of these facilities, standards for biodegradation in less controlled or uncontrolled conditions need more development. This is certainly the case for natural environments. Furthermore, home-composting conditions can be expected to vary greatly [139], even on a household-to-household basis. Since this is not an industrially regulated process, there is no confidence about the process conditions and the quality, concentration, and type of microorganisms in use. Additionally, independently from the adoption of bioplastics, home-composting also presents the risk of GHGs emissions [140,141] and therefore its sustainability might need a more thorough assessment.

One problem underlined in literature [35] is that many biodegradability standards require duplicates or at best triplicates of results. Triplicates are generally accepted in academia, where time and resources are often limited, and studies might be of a proof-of-concept nature. Still, the lack of reproducibility in academic studies is a serious topic of discussion [142]. Harmonized standards and official certifications have a great impact at international level and should represent the most reliable piece of information on a certain technical subject. Taking these considerations into account, the use of triplicates to assess statistical significance does not seem acceptable.

Finally, the scientific community, as well as regulatory agencies and certification companies, should consider the possibility that no standards will ever be able to encompass the diverse and dynamic conditions that are found in natural environments, and that producing standards claiming the opposite might mislead consumers on the actual environmental risks associated with plastic products.

### 4.3. Land and Water Use

One concern that has been expressed is the possible competition between the production of raw materials for the bio-based industry and agricultural production. *European Bioplastics* reports that, in the foreseeable future, bioplastics production will account for less than 0.02% of global agricultural area usage and therefore does not compete with the production of food. On the other hand, a Greenpeace report noted that it is important to consider where the land is situated and if it is concentrated within a few regions [143]. Furthermore, the report offered its own calculation on the effect of replacing plastic packaging with PLA packaging. The calculation suggests that in doing so, 32% of global annual corn production would be needed to be diverted to PLA, accounting for 1% of available agricultural land.

Furthermore, the production of bioplastics requires considerable use of fresh water, due to crop cultivation, e.g., corn farming intended for PLA production [144]. Water footprint analysis of bio-based plastics production is not often carried out in LCA studies, though some alarming results suggest that replacing European packaging production with bioplastics, would increase the related use of water to almost one fifth of the EU’s total freshwater withdrawal [18].

### 4.4. Issues Related to the Waste Disposal System

Europe has been greatly developing its composting capacity ever since the Landfill Directive 1999/31/EC. Still, the distribution of composting facilities at the national level needs to be considered. Looking at the Italian case, there are more than 330 composting facilities at the national level, but most are concentrated in the North of Italy [145]. The result is that compostable waste from the Center and the South needs to be transferred to the North, with additional costs and fuel consumption. Furthermore, if the resulting compost were to be sold in the South the opposite process would need to take place. If we are to adopt larger and larger volumes of compostable plastics, we need to be sure that composting facilities can cover this increase in volume at the regional level.

Biodegradable plastics are designed to completely break down due to microbial action but are also susceptible to degradation phenomena such as hydrolysis and thermal degradation, which generally play an important role in the biodegradation rate. As discussed, contamination of biodegradable plastics in common plastic recycling streams would be detrimental to the properties of the recyclates. The separation and sorting of this additional stream can also be complex and expensive [135]. Therefore, with an increasing use of biodegradable plastics, a reorganization in the recycling framework will be required, as well as informing consumers on the correct way to dispose of such products.

## 5. Conclusions

European governments are working towards a zero-emission economy, decoupled from fossil resources, and focused on sustainability and circularity. Reforming the way plastic products are produced, handled, and disposed of, is an important part of this process. The adoption of bio-based plastics might surely come with risks (use of fertilizers, social risks, etc.) as well as advantages, what seems certain is that it offers an alternative to fossil-based production and might therefore become a necessity in the future. Compostable or biodegradable plastics might result in problems if not sorted out from recycling streams. Still, applications like compost bags offer benefits since bag and content can be co-digested eliminating the need for separation and producing energy and compost. Materials should be designed to ensure effective degradation without causing technological issues while retaining their mechanical properties during the “use” phase. The use of biodegradable plastics to mitigate environmental pollution due to leakage in open environments is another discussed advantage, currently, this seems overly optimistic. Conditions in natural environments are dynamic, they greatly change within geographic regions and seasons, furthermore, the size and density of the plastic debris, as well as agglomeration with other materials, can influence the outcome. Additionally, the economic loss from this plastic waste is not solved by using biodegradable alternatives. In this sense, stricter regulations, promoting an environmental-friendly mentality, and investing in sustainability-focused education at an early age could prove to be a more effective use of resources.

To conclude, it can be expected that the bioplastics industry will receive incentives to grow, develop new technologies and materials, and scale-up its production to greater volumes. Once larger productions are achieved and larger volumes of bioplastics are circulated, more assessment will be undoubtedly required to understand the sustainability of these materials. However, one thing to keep in mind moving forward is that most of the issue lies in our throw-away mentality. Replacing one plastic with another, without replacing the mentality, is not a solution.

## Figures and Tables

**Table 1:** Lists of bioplastics and indication of bio-based origin and biodegradability. In the table, “y” means yes, “n” means no, and “y/n” refers to both statements being valid.

Polymer	Bio-Based	Biodegradable
Polylactic acid (PLA)	y	y
Starch blends, thermoplastic starch (TS)	y	y
Polyhydroxyalkanoates (PHAs)	y	y
Polybutylene succinate (PBS)	y/n	y
Polyurethanes (PURs)	y/n	y/n
Polycaprolactone (PCL)	n	y
Polyvinyl alcohol (PVA)	n	y
Polybutylene adipate terephthalate (PBAT)	n	y
Polyethylene Furanoate (PEF)	y	n
Bio-polypropylene (bio-PP)	y	n
Polytrimethylene terephthalate (PTT)	y	n
Bio-polyethylene terephthalate (bio-PET)	y	n
Bio-polyethylene (bio-PE)	y	n
Bio-polyamides (bio-PAs)	y	n

**Table 2:** List of common bio-based polymers and overview of their production.

Polymer	Technology Overview	Route
Polylactic acid	Fermentation of carbohydrates (e.g., starch) yields lactic acid which polymerizes to low M_n_ PLA. This depolymerizes to lactide, which polymerizes to high M_n_ PLA.	1
Polybutylene succinate	Bacterial fermentation of carbohydrates yields succinic acid, which is esterified to also obtain 1,4-butanediol. The two chemicals polymerize to PBS.	
Polyurethanes	Polyols obtained from plant oils are reacted with isocyanates or bio-isocyanates to yield PURs.	
Polyamides	Diacids derived from castor oil are reacted with a diamine to yield PAs. A typical pair is sebacic acid and decamethylenediamine (obtained from the acid).	
Polyethylene	Fermentation of saccharides yields bioethanol, then dehydrated to ethylene. Polymerization yields bio-PE.	
Thermoplastic starch	Typically obtained by gelatinization of starch (from corn, cassava, etc.) followed by casting or by extrusion of starch pellets and plasticizers.	2
Cellulose acetate	Cellulose from wood pulp is converted to a triacetate form which is then hydrolyzed to cellulose acetate.	
Regenerated cellulose	Cellulose is converted to a soluble form, then regenerated to obtain a film (cellophane) or a fiber (rayon).	
Polyhydroxyalkanoates	Intracellularly accumulated by different bacteria. Polyhydroxybutyrate was the first to be discovered.	3

**Table 3:** Main ISO and CEN standards relating to biodegradability and compostability of plastics.

Standard	Title
EN ISO 10210:2017	Plastics—Methods for the preparation of samples for biodegradation testing of plastic materials (ISO 10210:2012)
EN 14995:2006	Plastics—Evaluation of compostability—Test scheme and specifications
EN 13432:2000	Packaging—Requirements for packaging recoverable through composting and biodegradation—Test scheme and evaluation criteria for the final acceptance of packaging
EN 14046:2003	Packaging—Evaluation of the ultimate aerobic biodegradability of packaging materials under controlled composting conditions—Method by analysis of released carbon dioxide
EN 17033:2018	Plastics—Biodegradable mulch films for use in agriculture and horticulture—Requirements and test methods
ISO 17088:2012	Specifications for compostable plastics
EN ISO 14855-1:2012 EN ISO 14855-2:2018	Determination of the ultimate aerobic biodegradability of plastic materials under controlled composting conditions—Method by analysis of evolved carbon dioxide—Part 1: General method (ISO 14855-1:2012)—Part 2: Gravimetric measurement of carbon dioxide evolved in a laboratory-scale test (ISO 14855-2:2018)
EN ISO 16929:2019	Plastics—Determination of the degree of disintegration of plastic materials under defined composting conditions in a pilot-scale test (ISO 16929:2019)
EN ISO 20200:2015	Plastics—Determination of the degree of disintegration of plastic materials under simulated composting conditions in a laboratory-scale test (ISO 20200:2015)
ISO 23977-1:2020 ISO 23977-2:2020	Plastics—Determination of the aerobic biodegradation of plastic materials exposed to seawater—Part 1: Method by analysis of evolved carbon dioxide—Part 2: Method by measuring the oxygen demand in closed respirometer
EN ISO 14853:2017	Plastics—Determination of the ultimate anaerobic biodegradation of plastic materials in an aqueous system—Method by measurement of biogas production (ISO 14853:2016)
EN ISO 14851:2019	Determination of the ultimate aerobic biodegradability of plastic materials in an aqueous medium—Method by measuring the oxygen demand in a closed respirometer (ISO 14851:2019)
EN ISO 14852:2018	Determination of the ultimate aerobic biodegradability of plastic materials in an aqueous medium—Method by analysis of evolved carbon dioxide (ISO 14852:2018)
EN 17417:2020	Determination of the ultimate biodegradation of plastics materials in an aqueous system under anoxic (denitrifying) conditions—Method by measurement of pressure increase
EN ISO 10634:2018	Water quality—Preparation and treatment of poorly water-soluble organic compounds for the subsequent evaluation of their biodegradability in an aqueous medium (ISO 10634:2018)
EN ISO 14593:2005	Water quality—Evaluation of ultimate aerobic biodegradability of organic compounds in aqueous medium—Method by analysis of inorganic carbon in sealed vessels (CO_2_ headspace test) (ISO 14593:1999)
EN ISO 11733:2004	Water quality—Determination of the elimination and biodegradability of organic compounds in an aqueous medium—Activated sludge simulation test (ISO 11733:2004)
EN ISO 17556:2019	Plastics—Determination of the ultimate aerobic biodegradability of plastic materials in soil by measuring the oxygen demand in a respirometer or the amount of carbon dioxide evolved (ISO 17556:2019)
EN ISO 11266:2020	Soil quality—Guidance on laboratory testing for biodegradation of organic chemicals in soil under aerobic conditions (ISO 11266:1994)
EN ISO 15985:2017	Plastics—Determination of the ultimate anaerobic biodegradation under high-solids anaerobic-digestion conditions—Method by analysis of released biogas (ISO 15985:2014)
EN ISO 18830:2017	Plastics—Determination of aerobic biodegradation of non-floating plastic materials in a seawater/sandy sediment interface—Method by measuring the oxygen demand in closed respirometer (ISO 18830:2016)
EN ISO 19679:2020	Plastics—Determination of aerobic biodegradation of non-floating plastic materials in a seawater/sediment interface—Method by analysis of evolved carbon dioxide (ISO 19679:2020)
ISO 13975:2019	Plastics—Determination of the ultimate anaerobic biodegradation of plastic materials in controlled slurry digestion systems—Method by measurement of biogas production
ISO 22404:2019	Plastics—Determination of the aerobic biodegradation of non-floating materials exposed to marine sediment—Method by analysis of evolved carbon dioxide
ISO/DIS 23517-1 (under development)	Plastics—Biodegradable mulch films for use in agriculture and horticulture Part 1: Requirements and test methods regarding biodegradation, ecotoxicity and control of constituents

**Table 4:** List of CEN and ISO standards, technical reports and specifications, relevant to the life cycle assessment of bioplastics.

Standard	Title
EN 17228:2019	Plastics—Bio-based polymers, plastics, and plastics products—Terminology, characteristics and communication
EN 16760:2015	Bio-based products—Life Cycle Assessment
EN 16751:2016	Bio-based products—Sustainability criteria
EN 16575:2014	Bio-based products–Vocabulary
EN 16640:2017	Bio-based products—Bio-based carbon content—Determination of the bio-based carbon content using the radiocarbon method
EN 17351:2020	Bio-based products—Determination of the oxygen content using an elemental analyser
CEN/TR 16957:2016	Bio-based products—Guidelines for Life Cycle Inventory (LCI) for the End-of-life phase
CEN/TR 16721:2014	Bio-based products—Overview of methods to determine the bio-based content
CEN/TR 17341:2019	Bio-based products—Examples of reporting on sustainability criteria
CEN/TR 16208:2011	Biobased products—Overview of standards
EN ISO 14040:2006	Environmental management—Life cycle assessment—Principles and framework (ISO 14040:2006)
EN ISO 14044:2006	Environmental management—Life cycle assessment—Requirements and guidelines (ISO 14044:2006)
EN ISO 14046:2016	Environmental management—Water footprint—Principles, requirements and guidelines (ISO 14046:2014)
EN ISO 14067:2018	Greenhouse gases—Carbon footprint of products—Requirements and guidelines for quantification (ISO 14067:2018)
ISO/TS 14072:2014	Environmental management—Life cycle assessment—Requirements and guidelines for organizational life cycle assessment
ISO/TS 14048:2002	Environmental management—Life cycle assessment—Data documentation format
ISO/TS 14071:2014	Environmental management—Life cycle assessment—Critical review processes and reviewer competencies: Additional requirements and guidelines to ISO 14044:2006
ISO 14045:2012	Environmental management—Eco-efficiency assessment of product systems—Principles, requirements and guidelines
ISO/TR 14069:2013	Greenhouse gases—Quantification and reporting of greenhouse gas emissions for organizations—Guidance for the application of ISO 14064-1
ISO 22526-1:2020 ISO 22526-2:2020 ISO 22526-3:2020	Plastics—Carbon and environmental footprint of biobased plastics—Part 1: General principles—Part 2: Material carbon footprint, amount (mass) of CO_2_ removed from the air and incorporated into polymer molecule—Part 3: Process carbon footprint, requirements and guidelines for quantification
ISO 16620-1:2015 ISO 16620-2:2019 ISO 16620-3:2015 ISO 16620-4:2016 ISO 16620-5:2017	Plastics—Biobased content—Part 1: General principles—Part 2: Determination of biobased carbon content—Part 3: Determination of biobased synthetic polymer content—Part 4: Determination of biobased mass content—Part 5: Declaration of biobased carbon content, biobased synthetic polymer content and biobased mass content

**Table 5:** Impact category indicators in use in life cycle assessment.

Indicator	Units	Description
Global Warming Potential, GWP_100_	kg CO_2_ eq	Indicator of the potential global warming due to all greenhouse gas emissions over a period of 100 years; CO_2_ as reference
Ozone Depletion Potential, ODP	kg CFC-11 eq	Indicator of the potential destruction of the stratospheric ozone layer due to emissions; freon-11 as reference
Photochemical Ozone Creation Potential, POCP	kg ethene eq	Indicator of the photochemical ozone creation potential due to emission of gases; ethene as reference
Acidification Potential, AP	kg SO_2_ eq	Indicator of the potential acidification of water and soil due to emissions causing acid rain; sulphur dioxide as reference
Eutrophication, EU	kg PO_4_ eq	Indicator of the over-supply of nutrients to the ecosystem due to the release of nitrogen and phosphorous containing compounds which leads to algae bloom; phosphate as reference
Human Toxicity, HT	kg DCB eq	Indicator of the impact on human health due to toxic substances release; 1,4-dichlorobenzene as reference
Ecotoxicity, ET	kg DCB eq	Indicator of the impact on the ecosystem due to toxic substances release; 1,4-dichlorobenzene as reference
Land Use, LU	m^2^	Indicator of the land in use by the system under assessment
Water Use, WU	m^3^	Indicator of the water in use by the system under assessment
Abiotic Resource Depletion, ADP	kg Sb eq	Indicator of the depletion of non-living primary resources such as minerals and metals; antimony as reference
Abiotic Resource Depletion–Fossil fuels, ADP-fossil	MJ	Indicator of the fossil energy consumed by the system under assessment

**Table 6:** Summary of potential advantages and disadvantages related to the adoption of bioplastics.

Category	Description
Advantages of bioplastics	Reducing fossil fuel dependency by using renewable resources, replacing existing plastics with bio-based counterparts (e.g., drop-in plastics)
	Potential environmental benefits in terms of GWP reduction
	The use of compostable plastics, in applications where organic contamination is expected, simplifies waste management and returns carbon to soil as compost
	Anaerobic digestion of biodegradable plastics can produce large specific energy and contribute to achieve an optimal ratio of carbon to nitrogen in the process
	Biodegradable plastics could replace non-degradable plastics in products that are likely to leak in the environment, potentially mitigating plastic pollution
Disadvantages of bioplastics	High production costs and, possibly, lower performance than common plastics
	Lack of processability with common technologies or lack of know-how
	Small market volume does not justify major investments nor redesign of production frameworks and waste manager infrastructure
	Possible feedstock competition with biofuel and food industry
	Risk of fouling of recycling streams with biodegradable plastics
	Risk of landfilling biodegradable plastics resulting in GHG emissions
	Lack of dedicated composting and recycling infrastructure and logistics
	Uncertainty regarding biodegradability in different open environments
